# Course reassignment and learning engagement: the intervention strategy of AI-attributed reassignment

**DOI:** 10.3389/fpsyg.2026.1957576

**Published:** 2026-10-13

**Authors:** Han Li, Yuner Zhu, Yifan Tang

**Affiliations:** 1 School of Economics and Management, Huzhou Normal University, Huzhou, China; 2 School of Management, Fudan University, Shanghai, China

**Keywords:** AI-attributed reassignment, course reassignment, course selection, learning engagement, self-efficacy

## Abstract

**Introduction:**

Course reassignment is a common practice in university registration systems, yet reassigned students often exhibit lower learning engagement than those in self-selected courses, and the underlying psychological mechanism remains unclear. This study examined the impact of course reassignment on learning engagement, the mediating role of self-efficacy, and the buffering effect of AI-attributed course reassignment.

**Methods:**

Two scenario-based online experiments were conducted with university students randomly assigned to conditions. Experiment 1 (*N* = 393) used a single-factor between-subjects design comparing reassignment vs. self-selection. Experiment 2 (*N* = 293) added an AI-attributed reassignment condition in which an AI engine matched students' interests, traits, and abilities with the most suitable available courses (participants in the AI condition read a simulated AI-generated learning analysis report.). Participants completed a learning task and reported learning engagement and self-efficacy. Mediation analyses were conducted using bootstrapping.

**Results:**

Experiment 1 showed that course reassignment was associated with lower learning engagement compared with self-selection, and this effect was mediated by self-efficacy (indirect effect B = 0.198, SE = 0.051, 95%CI [0.096, 0.299]). Experiment 2 demonstrated that AI-attributed reassignment attenuated this negative effect. Learning engagement in the AI-attributed condition did not differ from self-selection and was significantly higher than in random reassignment. Moreover, the indirect effect through self-efficacy became non-significant (B = 0.140, SE = 0.113, 95% CI [−0.077, 0.362]).

**Discussion:**

These findings identify self-efficacy as a key psychological mechanism through which course reassignment impairs learning engagement, and demonstrate that AI-attributed reassignment can restore both self-efficacy and engagement by signaling person-course fit. This study extends self-efficacy theory to the course selection context and offers a feasible, technology-driven intervention for mitigating the negative consequences of course reassignment in higher education.

## 1 Introduction

Learning engagement, a core indicator of the quality of students' learning processes, directly influences the acquisition of knowledge and skills, the cultivation of innovative thinking, and the formation of lifelong learning abilities (Tang and Gao, 2025; Zhang and Yang, 2021; Lam et al., 2014). It has also been identified as a crucial safeguard for the quality of talent cultivation in higher education. However, considerable variation in learning engagement among students across universities may be attributed to differences in institutional course selection policies. Some universities adopt a course reassignment policy, which means a university's registration system will automatically assign a student to an open course section when the student's first-preference course is either fully enrolled or withdrawn due to low enrolment (Zhai et al., 2025; Yang, 2026). A substantially lower level of learning engagement has been observed among students in reassigned courses than among those in self-selected courses (Yang, 2026). Therefore, mitigating the decline in learning engagement attributable to course reassignment has become a key concern for both institutions and researchers.

Prior research has preliminarily examined the phenomenon of major or course reassignment and confirmed its negative impact on learning outcomes (Zhai et al., 2025; Yang, 2026). For instance, controlled major selection motivation has been associated with a significant reduction in major satisfaction (Nerona, 2021), and course reassignment has been linked to marked decreases in learning enjoyment and persistence (Malmberg et al., 2015). However, existing studies have largely been limited to phenomenon description or correlational analysis, and there is a lack of in-depth empirical investigation into the internal psychological mechanism through which course reassignment exerts its influence on learning engagement. In other words, the psychological mechanism underlying the effect of course reassignment on learning engagement has remained largely unclear, leaving intervention strategies in course reassignment contexts without a solid theoretical foundation.

The present study explored the impact of course reassignment on university students' learning engagement and its underlying psychological mechanism, and proposed intervention strategies to alleviate this negative effect. Through two experiments, we found that course reassignment (vs. self-selection) negatively affected learning engagement, with self-efficacy serving as the underlying psychological mechanism, and that this negative impact was significantly buffered by AI-attributed course reassignment. This study offers both theoretical contributions and practical implications. Theoretically, it reveals the psychological mechanism through course reassignment impairs learning engagement, thereby opening the black box of this influence, and identifies the mediating role of self-efficacy, thus broadening the application scope of self-efficacy theory. Practically, it proposes the intervention strategy of AI-attributed course reassignment, which provides a feasible approach for effectively mitigating the decline in learning engagement induced by course reassignment.

## 2 Literature review

Previous investigations into the antecedents of learning engagement have been conducted from two broad dimensions, namely individual psychological characteristics and external contextual factors. Specifically, at the individual level, cognitive and ability beliefs have been identified as critical predictors of learning engagement. Self-efficacy has been documented to exert a direct positive predictive effect, as well as indirect effects through pathways such as learning motivation and self-control (Yan et al., 2026; Wang et al., 2026). Factors including psychological resilience, mindfulness, and metacognition have also been found to correlate positively with learning engagement, with these effects often being mediated by enhanced self-esteem, improved self-regulation, or reduced negative emotions (Chen et al., 2025; Ge, 2025; Li et al., 2026; An et al., 2024). In terms of motivational drivers, autonomous motivation, career-planning clarity, and future self-continuity have all been shown to positively predict learning engagement (Zhong et al., 2025; Wang et al., 2026; Shen et al., 2024). A close relationship has also been established between emotional experiences and learning engagement. Learning engagement tends to be inhibited by negative emotions such as learning anxiety, academic burnout, and loneliness (Yang et al., 2026; Jin et al., 2025; Wang et al., 2024). Conversely, adverse childhood experiences and effort-reward imbalance have been found to indirectly undermine engagement by triggering negative affective states (Xu et al., 2026; Jin et al., 2025; Zhong et al., 2025).

At the external contextual level, social and interpersonal factors have been found to exert significant influences on learning engagement. Teacher autonomy support, emotional support, and teaching strategies have been shown to promote engagement by enhancing students' psychological capital, motivational beliefs, or social presence (Ma et al., 2026; Zheng and Pan, 2025; Kong et al., 2025; Zhang et al., 2024). In the family domain, positive prediction of learning engagement has also been observed for family support and parent-child relationships (Gao et al., 2021; Zhu et al., 2025). Important conditions for learning engagement have been further provided by curricular and technological environmental factors. Facilitation of engagement has been demonstrated for design principles of flipped classrooms, course clarity, and task relevance in online settings (Gu et al., 2022; Vo and Ho, 2024). Additionally, technological factors such as online presence, AI personalization, and perceived e-learning system quality have been found to exert positive effects (Pan and Lin, 2025; Jiang, 2026; Luo and Li, 2026). Moreover, inhibitory effects on learning engagement have been observed for controlling and depleting factors. Negative prediction of engagement by both mandatory digital learning and social media overload has been established, with this effect operating through increased cognitive load, triggered burnout, or induced ego depletion (Tang and Cui, 2025; Duan et al., 2025).

Despite the extensive body of research on the determinants of learning engagement, one commonly occurring contextual variable in higher education, course reassignment, has received little attention. The limited existing studies on reassignment have primarily focused on major reassignment and have mostly confined their examination to general learning outcomes such as academic achievement or satisfaction (Zhai et al., 2025; Yang, 2026; Nerona, 2021). Neither learning engagement as a core process variable nor its underlying psychological mechanisms have been systematically tested. Accordingly, the present study aims to examine the impact of course reassignment on students' learning engagement and to elucidate the mechanisms underlying this influence.

## 3 Hypothesis development

### 3.1 Course reassignment, self-efficacy, and learning engagement

Under course self-selection, students typically make decisions based on a comprehensive evaluation of their academic abilities, knowledge reserves, and personality preferences, and consequently a higher degree of compatibility between the selected courses and their existing ability levels and intrinsic personality traits is objectively more likely (Sadler-Smith, 2001). This compatibility is manifested in the alignment between course difficulty and students' knowledge structures, as well as in the correspondence between course content and their interest orientations. In contrast, under conditions of course reassignment (e.g., compulsory courses or reassignment), course content and difficulty are largely determined by external institutional arrangements and cannot be adjusted to match students' cognitive styles and affective inclinations (Assor et al., 2002). Course reassignment thus lowers students perceived compatibility between actual course requirements and their own ability structures and personality traits.

A lack of compatibility between personal abilities and course content can trigger negative appraisals of task difficulty at the cognitive level. When learning reassigned courses, students are more inclined to perceive learning tasks as externally imposed challenges that exceed their current capability thresholds (Thomas et al., 2016). The formation of self-efficacy beliefs is primarily influenced by four sources of information, namely, enactive mastery experiences, vicarious experiences, verbal persuasion, and physiological and emotional states (Bandura, 1997). Under conditions of course reassignment, the lack of intrinsic interest as a buffer makes it more difficult for students to derive positive affective experiences from the task itself, thereby weakening the internal drive to sustain positive efficacy judgments. Meanwhile, the passive acceptance of course arrangements make it difficult to establish effective cognitive connections between current learning tasks and past successful experiences, and to fully mobilize enactive mastery experiences. Due to the involuntary nature of course reassignment, viewing peers enrolled in the same course as referential models also becomes difficult, thus constraining the motivational effect of vicarious experiences. Furthermore, course reassignment (vs. self-selection) predisposes students to interpret anxiety arising during the learning process as a signal of personal inadequacy, rather than as a normal reaction to situational factors. Consequently, the weakening of these multiple sources of efficacy, including enactive mastery experiences, vicarious experiences, and physiological and emotional states, hampers the formation of positive judgments of personal competence, thereby significantly reducing perceived self-efficacy for completing learning tasks in such courses (Brady-Amoon and Fuertes, 2011).

In turn, according to self-efficacy theory (Bandura, 1997), individuals' persistence and willingness to exert effort when confronting difficulties are directly influenced by their level of perceived self-efficacy. Higher self-efficacy is associated with a greater likelihood of persevering and investing greater effort in the face of challenges, and with superior actual performance on difficult tasks, compared with lower self-efficacy (Zhao and Qin, 2021; Han et al., 2016). However, when low self-efficacy is induced by course reassignment, students tend to presuppose failure outcomes at the cognitive level, adopt avoidance strategies at the motivational level, and develop feelings of boredom and alienation at the emotional level (Assor et al., 2002). At the same time, goal commitment is also weakened by low self-efficacy (Bandura and Locke, 2003), such that students are more prone to give up rather than persist when encountering learning difficulties. Indeed, self-efficacy beliefs drive academic achievement primarily through the core pathways of goal setting and self-motivation (Zimmerman, 1992). These shifts in beliefs collectively suppress the willingness to engage in learning, as manifested in a reluctance to allocate sufficient time for deep thinking and in a lack of perseverance in overcoming learning difficulties. Therefore, the following hypotheses are proposed:

- *H1*: Course reassignment (vs. course self-selection) leads to lower learning engagement among university students.
- *H2*: This negative effect is mediated by perceived self-efficacy. Specifically, course reassignment negatively influences learning engagement by reducing perceived self-efficacy.

### 3.2 The intervention effect of course AI-attributed reassignment

The equations should be inserted in editable format from the equation editor. As discussed above, course reassignment reduces learning engagement by weakening perceived self-efficacy. However, if students still perceive a high degree of compatibility between their abilities or personality and the course to which they are finally assigned, the negative impact of course reassignment on perceived self-efficacy is attenuated. In other words, even under course reassignment, if students perceive a high degree of compatibility between the assigned course and their abilities or personality (Sadler-Smith, 2001), their perceived self-efficacy regarding that course does not decrease, and the negative impact of course reassignment on self-efficacy is thereby weakened. This inference suggests that the negative effect of course reassignment is not immutable but is contingent upon the establishment of a positive perception of course compatibility in contexts of passive acceptance. Furthermore, when subjectively perceived person–course fit is high, the negative impact of course reassignment on learning engagement is correspondingly attenuated or even eliminated. It can thus be inferred that enhancing perceived person–course fit may constitute a key direction for blocking the negative effect of course reassignment.

Given the potential of person–course fit to mitigate the negative effect induced by course reassignment, the decline in learning engagement caused by course reassignment may also be alleviated through the introduction of AI-attributed course reassignment. AI-attributed course reassignment refers to the practice of using an AI engine to collect and analyze students' interests, traits, and abilities (Sadler-Smith, 2001), so as to match them with the most suitable courses that still have vacancies, rather than assigning them randomly. In contrast to traditional random reassignment, although AI-attributed course reassignment does not alter reassignment (vs. self-selection) nature of course allocation, it provides students with assurance regarding the alignment between their course selections and their individual competencies. Therefore, even under AI-attributed course reassignment, the finally assigned course is likely to be perceived as highly compatible with personal characteristics such as abilities and personality. In turn, this high compatibility can mitigate the erosion of perceived self-efficacy by course reassignment, and thereby also alleviate the decline in learning engagement in the reassigned courses. Therefore, the following hypothesis is proposed:

- ***H3*:** AI-attributed course reassignment has an intervention effect on the negative effect of course reassignment (vs. self-selection) on learningengagement.

## 4 Experiment 1: the impact of course reassignment (vs. Self-selection) on learning engagement and the mediating role of self-efficacy

### 4.1 Research design

Experiment 1 examined the negative impact of course reassignment on learning engagement and the mediating role of self-efficacy, thereby testing Hypotheses H1 and H2. A single-factor, two-level (course selection: reassignment vs. self-selection) between-subjects design was adopted, with the course *Data Analysis in Daily Life* serving as the target course. Participants were randomly assigned to one of two conditions. In the self-selection condition, participants‘ preferences were described as leaning toward mathematical logic and data analysis, and they successfully enrolled in *Data Analysis in Daily Life*. In the reassignment condition, participants' preferences were described as leaning toward observation and life recording, and they selected *Smartphone Photography Skills*. However, they were then notified that the course was fully enrolled and that they had been compulsorily reassigned to *Data Analysis in Daily Life* with no possibility of alteration. A sample size estimation was conducted using G^*^Power (α = 0.05, power = 0.80, expected medium effect size *f* = 0.25). The minimum sample size required for a single-factor, two-level between-subjects design was calculated to be 128 participants.

### 4.2 Participants

A total of 400 university students were recruited from the data marketplace of the Credamo online experiment platform. Recruitment was restricted to individuals whose occupation was student, whose educational attainment was junior college or undergraduate, and whose age ranged from 17 to 24 years. A screening item (“Are you currently a full-time college student?”) was included at the beginning of the questionnaire. After excluding 7 participants who answered “no” to this screening question, 393 valid participants were retained (67.4%female; *M*_age_ = 20.02 years, *SD* = 2.01). The study was conducted in accordance with the ethical standards of the institutional review board, and informed consent was obtained from all participants prior to the experiment.

### 4.3 Measures

#### 4.3.1 Learning engagement

Participants‘ learning engagement was assessed using a 9-item scale adapted from Lam et al. (2014). This scale measures students' interest in and liking for learning and course. Sample items include “I am very interested in learning,” “I like what I am learning in this course,” and “I think learning is boring” (reverse-scored). Participants rated each item on a 5-point Likert scale ranging from 1 (*strongly disagree*) to 5 (*strongly agree*). The scale demonstrated good internal consistency in the current study (Cronbach's α = 0.857).

#### 4.3.2 Self-efficacy

Participants‘ self-efficacy was measured using a 6-item Academic Self-Efficacy Scale adapted from Roeser et al. (1996). This scale assesses students' beliefs that they can master the material and skills taught in school if given enough time and effort. Sample items include “ I'm certain I can master the skills taught in this course,” “ I can do even the hardest course work if I try,” and “ If I have enough time, I can do a good job on all my course work.” Participants rated each item on a 5-point Likert scale ranging from 1 (*not at all true of me*) to 5 (*very true of me*). The scale demonstrated good reliability in the current study (Cronbach's α = 0.931).

#### 4.3.3 Manipulation check

To verify the effectiveness of the course selection manipulation, participants completed a 2-item manipulation check adapted from Berry et al. (2018). The items are “I voluntarily chose to take this course” and “My choice to take this course was made freely.” Participants rated each item on a 7-point Likert scale ranging from 1 (*strongly disagree*) to 7 (*strongly agree*). The scale demonstrated high internal consistency (Cronbach's α = 0.970).

### 4.4 Procedure

The experiment was presented to participants as a public beta test of an online learning platform for general elective courses. All participants first read a cover story in which they were asked to imagine themselves as first-year students about to select courses for the first time, filled with anticipation for the forthcoming courses. They then browsed the descriptions of three simulated general elective courses: *Data Analysis in Daily Life, Smartphone Photography Skills*, and *Effective Interpersonal Communication*. Each course description was presented in a format imitating the interface of a university course selection system.

When participants made their course selection decision, different outcomes were presented by the system according to random assignment. In the self-selection group, participants‘ preferences were described as leaning toward mathematical logic and data analysis. Accordingly, they selected *Data Analysis in Daily Life* and received a message of successful enrollment. In the reassignment group, participants' preferences were described as leaning toward observation and life recording. They selected *Smartphone Photography Skills*, after which a notification was displayed indicating that the course was fully enrolled and that, according to the selection rules, they had been compulsorily reassigned to *Data Analysis in Daily Life*, with no possibility of alteration.

Thereafter, all participants gained access to the first session of *Data Analysis in Daily Life*, on the topic of correlation and causation. The learning material introduced a case study involving the relationship between ice cream sales and drowning incidents, and progressively explained the concepts of correlation, causation, and confounding variables. An open-ended reflection question was placed at the end of the material, asking participants to apply the acquired knowledge to judge the reasonableness of a conclusion regarding the relationship between coffee consumption and heart disease and to provide reasoning in no fewer than 20 words. This served as a behavioral indicator of learning engagement.

After the learning session, participants completed, in sequence, the measure of learning engagement, the measure of self-efficacy, and the manipulation check for course selection. Finally, they completed the attention check item and reported demographic information. Upon completion of the entire experiment, participants were fully debriefed regarding the study's purpose and were thanked for their participation.

### 4.5 Results

#### 4.5.1 Demographic characteristics

Demographic information for the reassignment and self-selection groups is presented in Table 1. Independent-samples *t*-tests and chi-square tests revealed no significant differences between the two groups in terms of gender, age, grade, major category, or monthly consumption (all *ps* > 0.05), indicating that random assignment produced equivalent groups.

**Table 1:** Comparison of demographic characteristics between the two groups.

Variable	Category	Reassignment (*n* = 194)	Self-selection (*n* = 199)	χ^2^/*t*	*P*
Gender	Male	65 (33.51%)	63 (31.66%)	0.153	0.696
	Female	129 (66.49%)	136 (68.34%)		
Age	M ± SD	20.06 ± 1.937	19.98 ± 2.078	0.354	0.724
Grade	Freshman	43 (22.16%)	52 (26.13%)	1.267	0.737
	Sophomore	51 (26.29%)	45 (22.61%)		
	Junior	47 (24.23%)	50 (25.13%)		
	Senior	53 (27.32%)	52 (26.13%)		
Major category	Literature	15 (7.73%)	17 (8.54%)	8.910	0.630
	Management science and engineering	14 (7.22%)	14 (7.04%)		
	Agriculture, forestry, animal husbandry and fishery	16 (8.25%)	13 (6.53%)		
	Education	19 (9.79%)	15 (7.54%)		
	Medicine and health	21 (10.82%)	18 (9.05%)		
	Law	10 (5.15%)	19 (9.55%)		
	History	20 (10.31%)	16 (8.04%)		
	Art	18 (9.28%)	19 (9.55%)		
	Economics	16 (8.25%)	13 (6.53%)		
	Science	12 (6.19%)	21 (10.55%)		
	Philosophy	15 (7.73%)	21 (10.55%)		
	Natural science	18 (9.28%)	13 (6.53%)		
Monthly consumption	Below 1,000 CNY	32 (16.49%)	35 (17.59%)	0.903	0.924
	1,000–1,500 CNY	42 (21.65%)	41 (20.60%)		
	1,500–2,000 CNY	51 (26.29%)	54 (27.14%)		
	2,000–3,000 CNY	41 (21.13%)	36 (18.09%)		
	Above 3,000 CNY	28 (14.44%)	33 (16.58%)		

Percentages are within-group proportions. For age, values are means ± standard deviations, and the test statistic is *t*. For all other categorical variables, values are frequencies and within-group percentages, and the test statistic is χ^2^.

#### 4.5.2 Manipulation check

A one-way ANOVA with course selection (reassignment vs. self-selection) as the independent variable and perceived autonomy as the dependent variable showed that the self-selection group perceived significantly higher course selection autonomy (*M* = 5.754, *SD* = 1.325) than the reassignment group (*M* = 3.340, *SD* = 1.610; *F*_(1, 391)_ = 264.014, *p* < 0.001, η^2^ = 0.403). Thus, the manipulation of course selection (reassignment vs. self-selection) was successful.

#### 4.5.3 Hypothesis testing

As shown in Figure 1, a one-way ANOVA with course selection (reassignment vs. self-selection) as the independent variable and the word count of the open-ended reflection responses as the dependent variable found that the word count was significantly higher in the self-selection group (*M* = 36.392, *SD* = 12.935) than in the reassignment group (*M* = 29.418, *SD* = 8.643; *F*_(1, 391)_ = 39.297, *p* < 0.001, η^2^ = 0.091). This provided preliminary evidence for the reduction in learning engagement caused by course reassignment.

**Figure 1:**
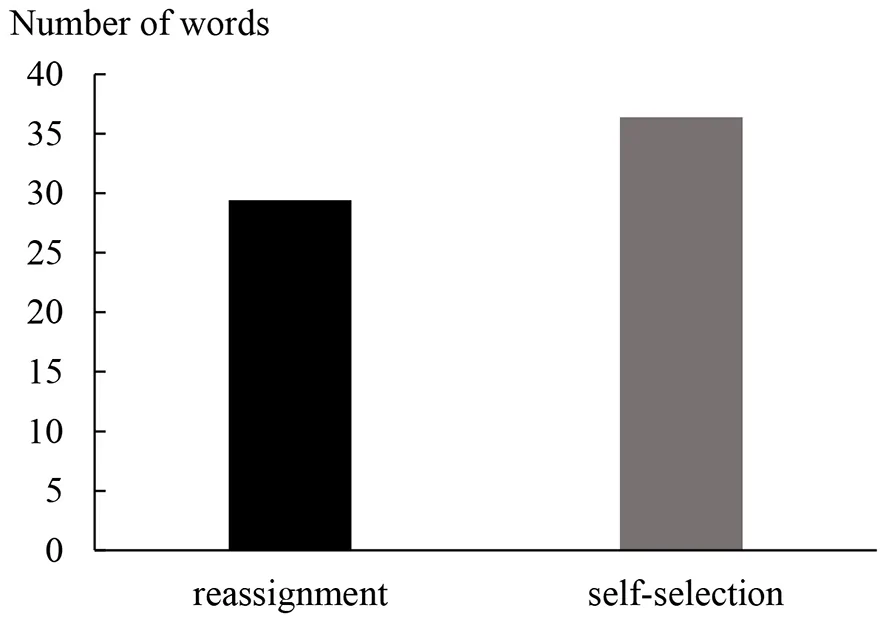
Number of words in open-ended reflection responses by course selection condition. Mean word counts are displayed for the reassignment and self-selection conditions. The self-selection condition produced a significantly higher word count than the reassignment condition, providing preliminary evidence that course reassignment may reduce learning engagement.

As shown in Figure 2, a one-way ANOVA with course selection (reassignment vs. self-selection) as the independent variable and the learning engagement scale score as the dependent variable found that the reassignment group reported lower learning engagement (*M* = 3.456, *SD* = 0.641) than the self-selection group (*M* = 3.829, *SD* = 0.678; *F*_(1, 391)_ = 31.343, *p* < 0.001, η^2^ = 0.074). Therefore, Hypothesis 1 was supported.

**Figure 2:**
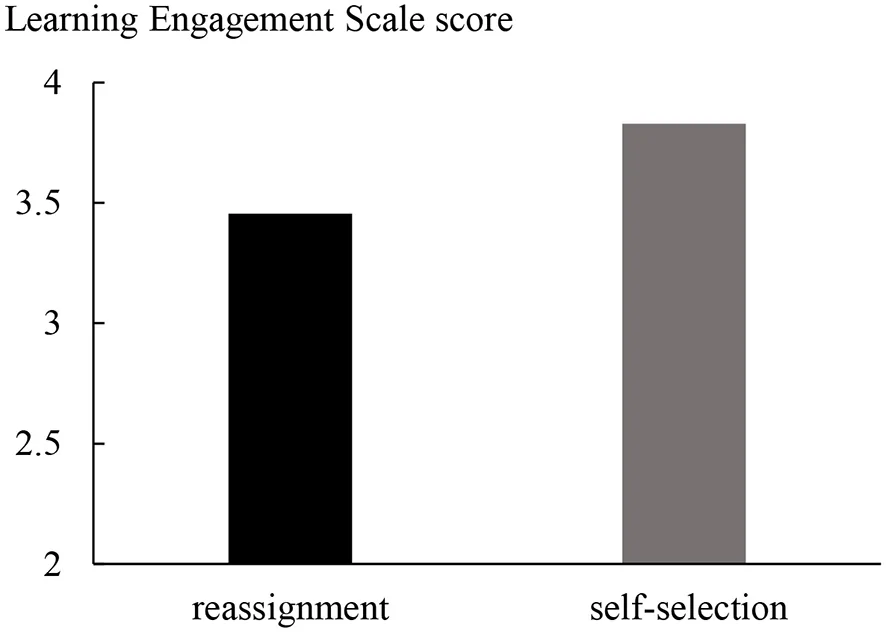
Learning engagement scale score by course selection condition. Mean scores are displayed for the reassignment and self-selection conditions. The self-selection condition produced a higher learning engagement score than the reassignment condition, indicating greater engagement in the self-selection condition.

Furthermore, a mediation analysis was performed using the bootstrap method (Model 4, 5000 samples; Hayes, 2013), with course selection (reassignment = 0, self-selection = 1) as the independent variable, self-efficacy as the mediator, and the learning engagement scale score as the dependent variable. The results showed that self-efficacy was significantly higher under self-selection than under reassignment (*B* = 0.284, *SE* = 0.074, *t* = 3.840, *p* < 0.001, 95%CI [0.139, 0.430]), and self-efficacy positively predicted learning engagement (*B* = 0.696, *SE* = 0.029, *t* = 24.149, *p* < 0.001, 95%CI [0.640, 0.753]). Moreover, a significant positive indirect effect of self-selection (vs. reassignment) on learning engagement through self-efficacy was observed (*B* = 0.198, *SE* = 0.051, 95%CI [0.096, 0.299]). Thus, the negative effect of course reassignment on learning engagement was mediated by self-efficacy, supporting Hypothesis 2.

#### 4.5.4 Discussion

Experiment 1 verified, through a scenario-based experiment, the negative impact of course reassignment on learning engagement and the mediating role of self-efficacy. The results indicated that the reduction in learning engagement attributable to course reassignment stems primarily from the weakening of perceived self-efficacy, which induces negative judgments regarding personal competence in course tasks. This finding reveals the psychological mechanism underlying the influence of course reassignment on learning engagement and provides a clear direction for subsequent intervention design. Given that the core of the problem lies in the decline of self-efficacy, restoring students' self-efficacy beliefs under conditions of reassignment becomes the next question to address. Building on this, Experiment 2 introduced AI-attributed reassignment to test whether this intervention can effectively compensate for the negative effect induced by reassignment.

## 5 Experiment 2: the intervention role of AI-attributed reassignment

### 5.1 Research design

Experiment 2 examined whether AI-attributed reassignment could buffer the negative impact of course reassignment on learning engagement, thereby testing Hypothesis H3. A single-factor, three-level (course selection: self-selection vs. reassignment vs. AI-attributed reassignment) between-subjects design was adopted, with the course *Data Analysis in Daily Life* serving as the target course. Participants were randomly assigned to one of three conditions. In the self-selection condition, participants' preferences were described as leaning toward mathematical logic and data analysis, and they successfully enrolled in *Data Analysis in Daily Life*. In the reassignment condition, participants' preferences were described as leaning toward observation and life recording, and they selected *Smartphone Photography Skills*. However, they were then notified that the course was fully enrolled and that they had been compulsorily reassigned to *Data Analysis in Daily Life* with no possibility of alteration. In the AI-attributed reassignment condition, participants' preferences were likewise described as leaning toward observation and life recording, and they also selected *Smartphone Photography Skills* before being informed that the course was full. However, they immediately received an AI-generated learning analysis report stating that, based on a pre-assessed cognitive style profile, their analytical thinking traits achieved an 85% match with the course's focus on data logic and critical thinking, and that this AI-driven matching had guided their reassignment to *Data Analysis in Daily Life*. A simulated radar chart and textual summary accompanied the report. A sample size estimation was conducted using G^*^Power (α = 0.05, power = 0.80, expected medium effect size *f* = 0.25). The minimum sample size required for a single-factor, three-level between-subjects design was calculated to be 159 participants.

### 5.2 Participants

A total of 300 university students were recruited from the data marketplace of the Credamo online experiment platform. Recruitment was restricted to individuals whose occupation was student, whose educational attainment was junior college or undergraduate, and whose age ranged from 17 to 24 years. A screening item (“Are you currently a full-time college student?”) was included at the beginning of the questionnaire. After excluding 7 participants who answered “no” to this screening question, the final sample comprised 293 valid participants (68.9% female; *M*_age_ = 19.93 years, *SD* = 1.90). The study was conducted in accordance with the ethical standards of the institutional review board, and informed consent was obtained from all participants prior to the experiment.

### 5.3 Measures

#### 5.3.1 Learning engagement

Learning engagement was measured using the same 9-item scale as in Experiment 1, adapted from Lam et al. (2014). Participants rated each item on a 5-point Likert scale ranging from 1 (*strongly disagree*) to 5 (*strongly agree*). The scale demonstrated good internal consistency in the current study (Cronbach's α = 0.954).

#### 5.3.2 Self-efficacy

Participants' self-efficacy was measured using the same 6-item Academic Self-Efficacy Scale as in Experiment 1, adapted from Roeser et al. (1996). Participants rated each item on a 5-point Likert scale ranging from 1 (*not at all true of me*) to 5 (*very true of me*). The scale demonstrated good reliability in the current study (Cronbach's α = 0.986).

#### 5.3.3 Manipulation check

To verify the effectiveness of the course selection manipulation, participants completed the same 2-item manipulation check as in Experiment 1, adapted from Berry et al. (2018). Participants rated each item on a 7-point Likert scale ranging from 1 (*strongly disagree*) to 7 (*strongly agree*). The scale demonstrated high internal consistency (Cronbach's α = 0.969).

### 5.4 Procedure

Participants were randomly assigned to one of three conditions: self-selection, reassignment, or AI-attributed reassignment. After browsing course descriptions and making a selection, participants in the self-selection group successfully enrolled in their chosen course, *Data Analysis in Daily Life*. Participants in the reassignment group were informed that their preferred course was full and were simply reassigned to *Data Analysis in Daily Life*. Participants in the AI-attributed reassignment group were similarly informed that their preferred course was full and were reassigned to *Data Analysis in Daily Life*, but they immediately received an AI-generated learning analysis report (a simulated AI-generated learning analysis report.), as described in the design section.

Thereafter, all participants studied the same micro-lecture as in Experiment 1. When the learning session ended, they completed the measures of learning engagement, self-efficacy, and the manipulation check for course selection, in that order. Finally, participants responded to an attention check item and provided demographic information. Upon completion of the entire experiment, participants were fully debriefed regarding the study's purpose and were thanked for their participation.

### 5.5 Results

#### 5.5.1 Demographic characteristics

Demographic information for the three groups is presented in Table 2. One-way ANOVA and chi-square tests revealed no significant differences among the three groups in terms of gender, age, grade, major category, or monthly consumption (all *ps* > 0.05), indicating that random assignment produced equivalent groups.

**Table 2:** Comparison of demographic characteristics among the three groups.

Variable	Category	Reassignment (*n* = 97)	Self-selection (*n* = 97)	AI-attributed reassignment (*n* = 99)	χ^2^/*F*	*P*
Gender	Male	29 (29.90%)	28 (28.87%)	34 (34.34%)	0.778	0.678
	Female	68 (70.10%)	69 (71.13%)	65 (65.66%)		
Age	M ± SD	20.05 ± 1.928	20.09 ± 1.899	19.65 ± 1.864	1.662	0.192
Grade	Freshman	26 (26.80%)	29 (29.90%)	31 (31.32%)	9.172	0.164
	Sophomore	31 (31.96%)	14 (14.43%)	23 (23.23%)		
	Junior	20 (20.62%)	29 (29.90%)	23 (23.23%)		
	Senior	20 (20.62%)	25 (25.77%)	22 (22.22%)		
Major category	Literature	10 (10.31%)	5 (5.15%)	8 (8.08%)	23.062	0.398
	Management science and engineering	7 (7.22%)	12 (12.37%)	8 (8.08%)		
	Agriculture, forestry, animal husbandry and fishery	10 (10.31%)	7 (7.22%)	8 (8.08%)		
	Education	10 (10.31%)	2 (2.06%)	7 (7.07%)		
	Medicine and health	6 (6.19%)	13 (13.40%)	12 (12.12%)		
	Law	12 (12.37%)	7 (7.22%)	5 (5.05%)		
	History	9 (9.28%)	6 (6.18%)	12 (12.12%)		
	Art	7 (7.22%)	11 (11.34%)	7 (7.07%)		
	Economics	7 (7.22%)	8 (8.25%)	9 (9.09%)		
	Science	6 (6.19%)	11 (11.34%)	5 (5.05%)		
	Philosophy	8 (8.24%)	8 (8.25%)	8 (8.08%)		
	Natural science	5 (5.14%)	7 (7.22%)	10 (10.11%)		
Monthly consumption	Below 1,000 CNY	16 (16.50%)	15 (15.46%)	15 (15.15%)	3.129	0.926
	1,000–1,500 CNY	18 (18.56%)	18 (18.56%)	17 (17.17%)		
	1,500–2,000 CNY	26 (26.80%)	31 (31.96%)	33 (33.34%)		
	2,000–3,000 CNY	24 (24.74%)	16 (16.49%)	19 (19.19%)		
	Above 3,000 CNY	13 (13.40%)	17 (17.53%)	15 (15.15%)		

Percentages are within-group proportions. For age, values are means ± standard deviations, and the test statistic is *F* (one-way ANOVA). For all other categorical variables, values are frequencies and within-group percentages, and the test statistic is χ^2^.

#### 5.5.2 Manipulation check

To verify participants' perceptions of autonomy across conditions, a one-way ANOVA was conducted. Results indicated a significant difference in perceived autonomy across conditions (*F*_(2, 290)_ = 243.565, *p* < 0.001, η^2^ = 0.627). Planned contrasts revealed that participants in the self-selection condition perceived significantly higher autonomy (*M* = 5.959, *SD* = 1.0574) than those in the reassignment condition (*M* = 2.716, *SD* = 1.1679; *F*_(1, 192)_ = 410.807, *p* < 0.001, η^2^ = 0.681) and those in the AI-attributed reassignment condition (*M* = 2.960, *SD* = 1.1924; *F*_(1, 194)_ = 346.602, *p* < 0.001, η^2^ = 0.641). The two reassignment conditions did not differ significantly from each other (*F*_(1, 194)_ = 2.078, *p* = 0.151). Thus, the course selection autonomy manipulation was successful.

#### 5.5.3 Effects on learning engagement

To examine the effect of course selection on learning engagement and determine if this effect is buffered when the reassignment is AI-attributed (H3), a one-way ANOVA was conducted. Results revealed a significant effect of condition on learning engagement (*F*_(2, 290)_ = 86.206, *p* < 0.001, η^2^ = 0.373). Planned contrasts were conducted to compare each of the reassignment conditions to the self-selection condition. These results are shown in Figure 3. The reassignment condition resulted in a significant decrease in learning engagement relative to the self-selection condition (self-selection: *M* = 3.658, *SD* = 0.970; reassignment: *M* = 2.294, *SD* = 0.998; *F*_(1, 192)_ = 93.065, *p* < 0.001, η^2^ = 0.326). However, when the reassignment was AI-attributed, it did not result in a decrease in learning engagement relative to self-selection (AI-attributed reassignment: *M* = 3.844, *SD* = 0. 705; *F*_(1, 194)_ = 2.378, *p* = 0.125). Further, a planned contrast directly comparing the two reassignment conditions confirmed that learning engagement was significantly greater for participants in the AI-attributed reassignment condition than for those in the reassignment condition (*F*_(1, 194)_ = 158.069, *p* < 0.001, η^2^ = 0.449). These results support H3.

**Figure 3:**
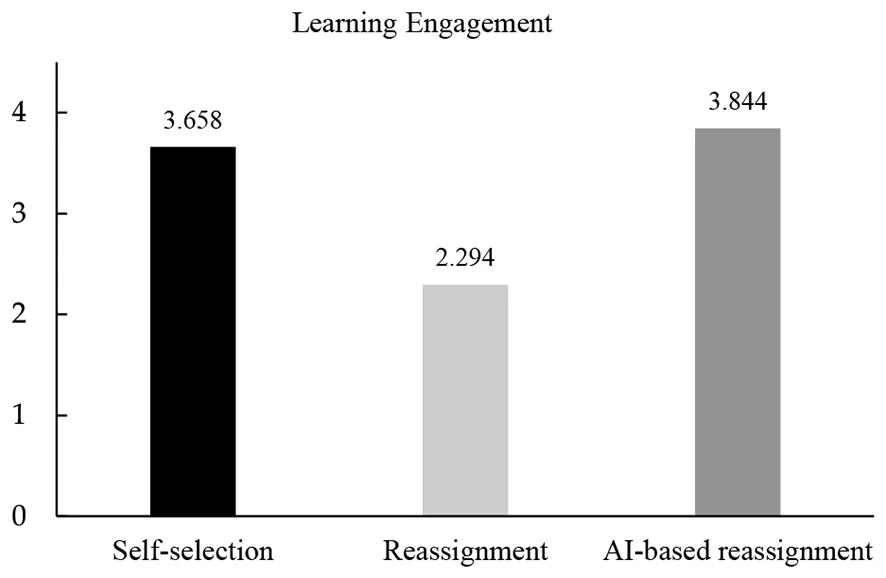
The intervention role of AI-attributed reassignment. Mean learning engagement scores are displayed for the self-selection, reassignment, and AI-attributed reassignment conditions. The AI-attributed reassignment condition restored learning engagement to a level comparable to that of self-selection and significantly higher than that of random reassignment, demonstrating the buffering effect of AI-driven matching.

#### 5.5.4 Indirect effects on learning engagement through self-efficacy

Given the mediating role of self-efficacy established in Experiment 1 and the buffering role of AI-attributed reassignment proposed in H3, a test of the indirect effects of reassignment and AI-attributed reassignment on learning engagement through self-efficacy was conducted using Model 4 of PROCESS with 5000 bootstrap samples (Hayes, 2013). Prior to the mediation analysis, two predictors were created using indicator coding (Hayes and Preacher, 2014), with the self-selection condition serving as the reference group (self-selection = 0, reassignment = 1, AI-attributed reassignment = 2). Replicating the mediation pattern of Experiment 1, the negative indirect effect of reassignment (vs. self-selection) on learning engagement through self-efficacy was significant (*B* = −1.109, *SE* = 0.123, 95% CI [-1.372,−0.867]). This significant indirect effect occurred because reassignment reduced self-efficacy (*B* = −1.330, *SE* = 0.139, *t* = −9.565, *p* < 0.001, 95%CI [−1.604, −1.056]), and self-efficacy positively predicted learning engagement (*B* = 0.834, *SE* = 0.024, *t* = 34.635, *p* < 0.001, 95%CI [0.787, 0.881]). In contrast, the indirect effect of AI-attributed reassignment (vs. self-selection) through self-efficacy was non-significant (*B* = 0.140, *SE* = 0.113, 95%CI [−0.077, 0.362]). These results indicate that AI-attributed reassignment buffers the negative effect of course reassignment on learning engagement by restoring self-efficacy. When the reassignment was framed as AI-driven matching, the indirect pathway through self-efficacy was was no longer statistically detectable. These findings support H3.

### 5.6 Discussion

Experiment 2 adopted a single-factor, three-level design to directly compare the effects of reassignment and AI-attributed reassignment against self-selection. The findings indicate that while reassignment has a clear negative effect on learning engagement, which is mediated by reduced self-efficacy, the AI-attributed reassignment condition attenuates this detrimental effect. Specifically, when the course reassignment was framed as the result of AI-driven personalized matching, participants' self-efficacy and learning engagement were restored to levels comparable to those observed in the self-selection condition. This suggests that it is not the act of reassignment *per se* that damages self-efficacy, but rather the absence of a perceived rationale linking the assigned course to the student's personal competencies.

## 6 General discussion

### 6.1 Conclusions

Grounded in self-efficacy theory, the present research investigated the mechanism through which course reassignment (vs. self-selection) influences learning engagement and the intervening role of AI-attributed course reassignment via two online experiments. Experiment 1 used a scenario-based design to examine the negative effect of course reassignment on learning engagement and revealed that self-efficacy mediated this relationship. Experiment 2 introduced AI-attributed course reassignment as a intervention tool, replicated the association between course reassignment and learning engagement, and further demonstrated that AI-attributed reassignment can effectively compensate for the detrimental effect of reassignment. Specifically, the learning engagement of participants in the AI-attributed reassignment condition did not differ significantly from that of participants in the self-selection condition, and both were significantly higher than that in the random reassignment condition. Taken together, these two experiments indicate that course reassignment was associated with lower learning engagement, and that AI-attributed reassignment buffers this negative effect by restoring students' self-efficacy beliefs.

### 6.2 Theoretical contributions

First, this research tested the main effect of course reassignment (vs. self-selection) on learning engagement, thereby enriching the literature on course reassignment. Although prior studies have documented the negative impact of reassignment on learning outcomes (Zhai et al., 2025; Yang, 2026), most of these studies were conducted in the context of admission reassignment (e.g., major reassignment) rather than in the post-enrollment course selection context. The present study found that even course reassignment can have a negative effect on students' short-term learning engagement.

Second, this research uncovered the mediating role of self-efficacy between course reassignment (vs. self-selection) and learning engagement, thereby opening the “black box” of the psychological mechanism through which reassignment impairs learning engagement. Existing studies have focused primarily on the direct effect of choice on learning outcomes (Zhai et al., 2025; Yang, 2026), overlooking the changes that occur in students' judgments of their own capabilities during this process. Grounded in self-efficacy theory, the present research finds that reassignment damages learning engagement precisely because it weakens students' confidence in their ability to master course tasks, leading them to underestimate their competence and anticipate failure. This finding offers a new theoretical lens for understanding the academic difficulties faced by reassigned students.

Finally, this research identified the interventional mechanism of AI-attributed reassignment. Prior research has indicated that the formation and enhancement of self-efficacy rely primarily on mastery experiences, vicarious experiences, and other direct or indirect success experiences (Bandura, 1997; Byars-Winston et al., 2017). The present research demonstrates that, in reassignment situations, AI technology can convey information about the fit between a course and students' individual attributes, thereby enhancing their perceived fit and restoring their impaired self-efficacy beliefs. This suggests that, beyond personal success experiences and role modeling, technology-based personalized matching information can serve as an effective pathway for sustaining self-efficacy, offering a new theoretical direction for intervention design in reassignment contexts.

### 6.3 Practical implications

First, greater attention should be paid to restoring the self-efficacy of reassigned students. University administrators and instructors should recognize that the core challenge faced by reassigned students lies not merely in a mismatch with course content, but in a lack of confidence in their own ability to succeed. When students underestimate their competence and anticipate failure, their learning engagement is unlikely to be genuinely stimulated, even if the course content is highly practical and the teaching is excellent. Therefore, teaching management should focus on helping students build a sense of competence regarding course tasks, rather than solely emphasizing the importance or utility of the course. For instance, during the course orientation phase, instructors can design moderately difficult introductory tasks that enable students to gain early success experiences and gradually develop positive judgments of their own abilities.

Second, AI technology should be leveraged to achieve personalized course matching. The present research demonstrates that AI-attributed course reassignment, in which an AI engine collects and analyzes students' interests, traits, and abilities to match them with the most suitable available courses, can effectively buffer the decline in learning engagement, raising it to a level comparable to that of self-selection and significantly higher than that of random assignment. Moreover, even when the course allocation system itself cannot be changed, a low-cost, scalable intervention can still be implemented: providing reassigned students with an AI-generated person–course fit report that reveals the internal consistency between the student and the course. This report significantly enhances self-efficacy and, in turn, learning engagement. In other words, it helps rebuild students' confidence in their abilities through technological means without requiring modifications to the existing allocation infrastructure.

Third, the psychological needs of reassigned students should be addressed. Instructors are advised to provide additional support to reassigned students, helping them gradually accumulate success experiences to boost self-efficacy. In classroom interactions, teachers can purposefully pose questions of increasing difficulty so that reassigned students can progressively experience a sense of competence. In assignment design, tiered task options can be provided, allowing students to choose an appropriate level of challenge based on their own ability and to accumulate mastery experiences as they complete the tasks. In this way, students can gradually build confidence in their ability to handle learning tasks, which in turn fosters intrinsic learning engagement.

### 6.4 Limitations and future directions

First, this research examined only the mediating mechanism of self-efficacy based on self-efficacy theory. However, it is likely that the influence of course reassignment on learning involves other mediating pathways. For example, reassignment may affect learning outcomes by undermining intrinsic motivation or through other psychological processes. Future research should investigate additional possible mechanisms in order to reveal the negative effects of reassignment more comprehensively.

Second, this research tested only AI-attributed reassignment as a compensatory intervention, yet the pathways for restoring self-efficacy are not limited to this single approach. Future studies could compare the compensatory effects of different efficacy-enhancement strategies (e.g., providing success experiences, role modeling, and positive feedback) or non-technological approaches (e.g., teacher guidance and peer support) on the negative consequences of reassignment, and explore the relative advantages and boundary conditions of each strategy.

Third, the experimental manipulation in both studies confounded the act of reassignment with interest-content mismatch. In the reassignment condition, participants were told their preferences leaned toward observation and life recording but were assigned to a data analysis course. Consequently, the observed negative effects may partly or wholly reflect the mismatch between assigned course content and stated interests, rather than the loss of choice *per se*. This confound limits the causal interpretation of the reassignment effect. Future research should adopt a fully crossed 2 (choice vs. assignment) × 2 (fit vs. misfit) design to isolate the unique contributions of choice deprivation and interest mismatch.

Third, although the present research demonstrates that AI-attributed reassignment can effectively compensate for the negative impact of reassignment, this effect may be subject to boundary conditions. For instance, factors such as students' trust in AI technology may influence the effectiveness of the intervention, and these warrant further investigation. In addition, we recommend that future research include a validated measure of perceived person–course fit to examine the full serial mediation pathway.

Finally, this research primarily relied on online scenario-based experiments and measured participants' willingness to engage in learning rather than their actual classroom learning behaviors, which limits the external validity of the findings. Future research should conduct field experiments in authentic teaching contexts to enhance the generalizability of the conclusions.

## Data Availability

The raw data supporting the conclusions of this article will be made available by the authors, without undue reservation.
